# Escherichia ruysiae May Serve as a Reservoir of Antibiotic Resistance Genes across Multiple Settings and Regions

**DOI:** 10.1128/spectrum.01753-23

**Published:** 2023-06-15

**Authors:** Edgar I. Campos-Madueno, Claudia Aldeia, Parham Sendi, Andrea Endimiani

**Affiliations:** a Institute for Infectious Diseases (IFIK), University of Bern, Bern, Switzerland; b Graduate School of Cellular and Biomedical Sciences, University of Bern, Bern, Switzerland; JMI Laboratories

**Keywords:** animal, CTX-M-15, ESBL, ST5792, environment

## Abstract

Gut colonization with multidrug-resistant *Enterobacterale*s (MDR-*Ent*) has reached worrisome levels worldwide. In this context, Escherichia ruysiae is a recently described species mostly found in animals. However, its spread and impact on humans is poorly understood. A stool sample from a healthy individual living in India was screened for the presence of MDR-*Ent* using culture-based methods. Colonies were routinely identified using matrix-assisted laser desorption ionization–time of flight mass spectrometry (MALDI-TOF MS) and phenotypically characterized by broth microdilution. Illumina and Nanopore whole-genome sequencing (WGS) platforms were implemented to generate a complete assembly. *E. ruysiae* genomes deposited in international databases were used for a core genome phylogenetic analysis. An extended-spectrum β-lactamase (ESBL)-producing E. coli strain (S1-IND-07-A) was isolated from the stool. WGS confirmed that S1-IND-07-A was indeed *E. ruysiae*, belonged to sequence type 5792 (ST5792), core genome (cg) ST89059, serotype O13/O129−:H56-like, clade IV phylogroup, and possessed five virulence factors. A copy of *bla*_CTX-M-15_ and five other antimicrobial resistance genes (ARGs) were detected in a conjugative IncB/O/K/Z plasmid. A database search identified 70 further *E. ruysiae* strains from 16 countries (44, 15, and 11 strains isolated from animals, the environment, and humans, respectively). The core genome phylogeny revealed five major STs: ST6467, ST8084, ST2371, ST9287, and ST5792. Three out of the seventy strains possessed important ARGs: OTP1704 (*bla*_CTX-M-14_; ST6467), SN1013-18 (*bla*_CTX-M-15_; ST5792), and CE1758 (*bla*_CMY-2_; ST7531). These strains were of human, environmental, and wild animal origin, respectively. *E. ruysiae* may acquire clinically important ARGs and transmit them to other species. Due to its zoonotic potential, further efforts are needed to improve routine detection and surveillance across One Health settings.

**IMPORTANCE** Escherichia ruysiae is a recently described species of the cryptic clades III and IV of the genus Escherichia and is commonly found in animals and the environment. This work highlights the zoonotic potential of *E. ruysiae*, as it has been shown to colonize the human intestinal tract. Importantly, *E. ruysiae* may be associated with conjugative plasmids carrying clinically relevant antibiotic resistance genes. Therefore, it is important to closely monitor this species. Overall, this study highlights the need for improved identification of Escherichia species and continued surveillance of zoonotic pathogens in One Health settings.

## INTRODUCTION

The genus Escherichia is comprised of 3 monophyletic species (Escherichia albertii, E. coli, and E. fergusonii), including cryptic clades I, II, III and IV (E. ruysiae), and V (E. marmotae) (1–3). Furthermore, E. coli *sensu stricto* can be subdivided into 7 phylogroups (A, B1, B2, C, D, E, and F) corresponding to specific host/source niches where this pathogen can be found, such as commensal E. coli of the gastrointestinal tract in humans (groups A or B1) and animals (group B1) and the environment (e.g., wastewater; B2 or D) (4). On this note, in humans, E. coli can cause extraintestinal infections due to specific virulence factors, and treatment of these infections can be challenging because of the possible presence of antibiotic resistance genes (ARGs), such as those encoding extended-spectrum β-lactamases (ESBLs; e.g., *bla*_CTX-Ms_), which compromise the treatment (5).

*E. ruysiae* has been recently reported from animal and environmental sources, but it is still very rarely isolated in humans (1, 6, 7). Moreover, routine identification and differentiation from its counterpart E. coli is very complex using standard laboratory methods (e.g., matrix-assisted laser desorption ionization–time of flight mass spectrometry [MALDI-TOF MS]), unless a PCR-based approach (e.g., targeting the *chuA* and *aes* genes) or whole-genome sequencing (WGS) are used (1, 2, 8, 9). Overall, very little is known about the role of *E. ruysiae* in humans, in particular about its potential to spread ARGs. Of note, a plasmid-associated (IncI) *bla*_CTX-M-14_-possessing Escherichia cryptic clade IV strain (OPT1704) was recently isolated from the stool of a traveler to Asia (10). This strain was later characterized and proposed as *E. ruysiae* sp. nov., which also includes cryptic clade III (1).

In this work, we characterized a *bla*_CTX-M-15_-harboring *E. ruysiae* strain (S1-IND-07-A) isolated from a fecal sample of a healthy individual living in India. To investigate the spread of *E. ruysiae* in humans, we searched for other *E. ruysiae* genomes in international databases and compared the results using core genome phylogenetic analysis. Furthermore, we investigated the distribution of the associated ARGs, plasmids, virulence factors, and serotypes characteristic of strains isolated from various human and nonhuman samples.

## RESULTS AND DISCUSSION

The first aim of this study was to characterize a rare ESBL-producing *E. ruysiae* strain isolated from a person living abroad. To contextualize our findings, the second aim was to search global databases for other reported *E. ruysiae* strains isolated from different sources to perform a core genome phylogeny analysis. Finally, we examined the associated ARGs, plasmid replicons, virulence, and serotype distribution of all *E. ruysiae* strains included in this study to evaluate the potential of *E. ruysiae* as a human pathogen.

### Strain isolation and phenotypic testing.

Screening of the individual’s stool yielded a third-generation cephalosporin-resistant strain (S1-IND-07-A) that was identified by MALDI-TOF MS as E. coli (score, 2.56). As shown in Table 1, phenotypic testing indicated that the strain was resistant to cephalosporins (e.g., cefotaxime MIC of 16 μg/mL), fluoroquinolones (e.g., ciprofloxacin MIC of 1 μg/mL), and trimethoprim-sulfamethoxazole (MIC, >4/76 μg/mL).

**TABLE 1:** Antimicrobial susceptibility testing results of *E. ruysiae* strain S1-IND-07-A, the E. coli J53d-R1 transconjugant, and the original E. coli J53d-R1 recipient

Antibiotic	MIC value (μg/mL) for strain:^*a*^		
	*E. ruysiae* S1-IND-07-A	E. coli J53d-R1 TC-S1-IND-07-A	E. coli J53d-R1
Piperacillin-tazobactam	≤8/4 (S)	≤8/4 (S)	≤8/4 (S)
Ticarcillin-clavulanate	≤16/2 (S)	≤16/2 (S)	≤16/2 (S)
Ceftazidime	2 (S)	2 (S)	≤1 (S)
Cefotaxime	16 (R)	16 (R)	≤1 (S)
Cefepime	≤1 (S)	≤1 (S)	≤1 (S)
Aztreonam	4 (S)	4 (S)	≤2 (S)
Imipenem	≤1 (S)	≤1 (S)	≤1 (S)
Meropenem	≤1 (S)	≤1 (S)	≤1 (S)
Doripenem	≤0.12 (S)	≤0.12 (S)	≤0.12 (S)
Ertapenem	≤0.25 (S)	≤0.25 (S)	≤0.25 (S)
Gentamicin	≤1 (S)	≤1 (S)	≤1 (S)
Tobramycin	≤1 (S)	≤1 (S)	≤1 (S)
Amikacin	≤4 (S)	≤4 (S)	≤4 (S)
Ciprofloxacin	1 (R)	0.5 (S)	≤0.25 (S)
Levofloxacin	2 (R)	≤1 (S)	≤1 (S)
Colistin	≤0.25 (S)	≤0.25 (S)	≤0.25 (S)
Polymyxin B	≤0.25 (S)	≤0.25 (S)	≤0.25 (S)
Doxycycline	16 (NA)	16 (NA)	≤2 (NA)
Minocycline	≤2 (NA)	4 (NA)	≤2 (NA)
Tigecycline	≤0.25 (S)	≤0.25 (S)	≤0.25 (S)
Trimethoprim-sulfamethoxazole	>4/76 (R)	>4/76 (R)	≤0.5/9.5 (S)

a R, resistant; S, susceptible; NA, not available. MICs were interpreted according to the 2023 EUCAST breakpoints for *Enterobacterales* (v13.0).

### Resistome, serotype, and virulence characterization.

Using WGS, the species was confirmed as *E. ruysiae* (average nucleotide identity using BLAST [ANIb], 98.67% and 91.92% compared to *E. ruysiae* OPT1704 and E. coli DSM 30083, respectively). This was also supported by *in silico* phylotyping, which determined that the species belonged to Escherichia cryptic clade IV.

S1-IND-07-A was of sequence type 5792 (ST5792) and core genome (cg) ST89059. Its genome consisted of a 4.4-Mb chromosome and 2 circular plasmids of 105.8 kb (IncB/O/K/Z) and 1.5 kb [Col(MG828)]. Most ARGs were carried by the IncB/O/K/Z plasmid (p1-S1-IND-07-A): *bla*_CTX-M-15_, *aadA1*, *dfrA1*-like (99.79% identity), *qnrS1*, *sul2*, and *tet*(A). In the chromosome, mutations encoding for the quinolone resistance-determining region substitution (S83A) in GyrA were identified, together with the *mdf*(A)-like ARG (91.32% identity) (see Table S1 in the supplemental material). Overall, the genotypic screening results were consistent with the observed phenotype (Table 1).

Last, *in silico* serotyping revealed that S1-IND-07-A carried O13/O129-like (*wzx* or *wzy*; 99.52% identity) and H56-like (*fliC*; 92.45% identity) antigens. Several virulence factor genes were also identified, including those encoding colonization factors (*fimH*), fitness (*iutA* and *kpsMII*), and toxins (*pic* and *sat*) (Table S1). Of note, p1-S1-IND-07-A carried the colicin Ib virulence factor gene *cib*, which is known to enhance virulence (i.e., killing colicin-susceptible bacteria) in, for example, E. coli and Salmonella enterica (Table S1) (11–13).

### Core genome phylogeny of *E. ruysiae* strains derived from human and nonhuman sources.

To investigate the origin of S1-IND-07-A, we performed a database search for other *E. ruysiae* strains of the same sequence type and core genome sequence type (ST5792 and cgST89059). We also included strains isolated from different sources to identify potential ARG hot spots and evaluate the pathogenic potential of *E. ruysiae* in humans. Overall, we included a total of 70 *E. ruysiae* genomes with complete metadata (collection date, country of isolation, host, sample/BioSample accession number) from the NCBI (*n* = 13: clade III, *n* = 9; clade IV, *n* = 4) and Enterobase (*n* = 57: clade III, *n* = 27; clade IV, *n* = 30) databases.

**FIG 1:**
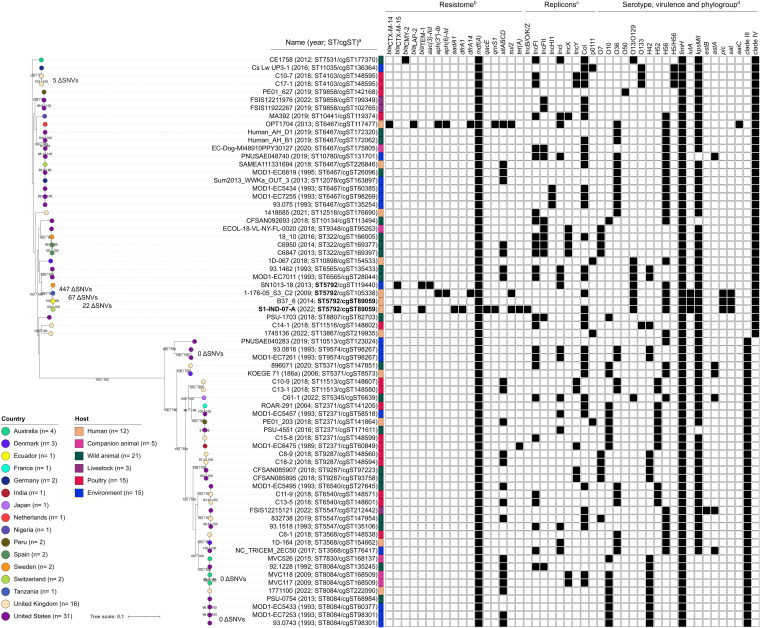
Core genome phylogeny of *E. ruysiae* cryptic clades III and IV (*n* = 71). In the maximum likelihood tree, the country and host are represented by colored circles and squares, respectively. SNV differences between S1-IND-07-A (bold) and other ST or cgSTs (bold) are represented by a ΔSNV. Bootstrap values are shown on branches (SH-aLRT and UFboot, respectively). The tree scale represents the average number of nucleotide substitutions per site. *^a^*The name corresponds to the sample, strain, or other unique identifier as per the BioSample description in NCBI or Enterobase. *^b^*Antibiotic and disinfectant genes. *^c^*Replicon sequences are shown by incompatibility group. *^d^*O and H antigen closest matches; representative virulence factors (colonization [*fimH*], fitness [*iutA* and *kpsMII*], toxins [*estB*, *astA*, *pic*, and *sat*], and effectors [*aaiC*]); clades III and IV correspond to cryptic clades. See Tables S2 and S4 for the figure metadata and SNV matrix, respectively.

As a result, along with our strain, we identified a total of 71 *E. ruysiae* strains belonging to the cryptic clades III (*n* = 36) and IV (*n* = 35), detected between 1989 and 2022 (Fig. 1; Table S2). The strains were isolated from animal (*n* = 44; 62%), environmental (*n* = 15; 21%), and human sources (*n* = 12; 17%). The strains originated from 16 countries, mainly from the United States (*n* = 31) and the United Kingdom (*n* = 16).

A total of 30 unique STs were identified, five of which consisted of larger groups of STs: ST6467 (*n* = 9), ST8084 (*n* = 8), ST2371 (*n* = 6), ST9287 (*n* = 4), and ST5792 (*n* = 4) (Fig. 1; Table S2). In particular, three strains (B37_6, 1-176-05_S3_C2, and SN1013-18) belonging to the same ST as S1-IND-07-A (ST5792) were detected. Strain B37_6 also belonged to cgST89059 (like S1-IND-07-A), suggesting a closely related lineage (22 single nucleotide variant differences [ΔSNVs]), in contrast to the other two strains (67 to 447 ΔSNVs) (Fig. 1; Table S4). Of note, strain B37_6, originally identified as E. coli, was isolated from a diarrheal stool sample of an adult woman in a 2014 study in Ecuador (14).

In addition to cgST89059, four other sporadic cgST groups (cgST148595, cgST168509, cgST98267, and cgST98301) were observed. Overall, they consisted of strains isolated from poultry in the UK (C17-1 and C10-7), companion animals in Australia (MVC117 and MVC118), and the environment in the United States (MOD1-EC7261, 93.0816, MOD1-EC7253, and 93.0743) (Fig. 1; Table S4) (6, 15–17).

As depicted in Fig. 1, in addition to S1-IND-07-A, three other *E. ruysiae* strains from clade IV carried β-lactamase (*bla*) genes: CE1758 (*bla*_CMY-2_), OPT1704 (*bla*_CTX-M-14_ and *bla*_LAP-2_), and SN1013-18 (*bla*_CTX-M-15_ and *bla*_TEM-1_). The *bla*_CTX-M-14_ and *bla*_LAP-2_ genes in OPT1704 were associated with an IncI plasmid, while the *bla*_CMY-2_, *bla*_CTX-M-15_, and *bla*_TEM-1_ genes in CE1758 and SN1013-18 were unspecified (1, 18).

Most of the other ARGs were detected only in S1-IND-07-A, SN1013-18, OPT1704, and 1-176-05_S3_C2, while the rest of the strains carried only *mdf*(A) and (sporadically) *sitABCD*. Furthermore, many replicon sequence types were detected in both clades III and IV, most frequently Col and IncFI type (Fig. 1; Table S2). These data suggest that *E. ruysiae* can be associated not only with resistance plasmids but also with many other plasmid backbones that have the potential to acquire ARGs (19).

A wide range of serotypes were identified, but none of them were perfect matches, as seen in S1-IND-07-A carrying O13/O129-like:H56-like (Fig. 1; Table S1). Overall, clade III strains were more often O10-like (*n* = 16) or O36-like (*n* = 10) and H42-like (*n* = 15), H52-like (*n* = 11) and H56-like (*n* = 10), while clade IV strains were O36-like (*n* = 11) or O13/O129-like (*n* = 8) and H56-like (*n* = 20). Furthermore, many virulence factors were also detected, but only a few were clinically relevant, as they were related to host colonization (*fimH*), fitness (*iutA* and *kpsMII*), toxins (*estB*, *astA*, *pic*, abd *sat*), and effectors (*aaiC*) (20).

Notably, all strains carried *fimH*-type variants, while 4 strains were positive for both *estB* and *astA* (pig strain FSIS12215121) and *pic* and *sat* (human strains 1–176-05_S3_C2, B37_6, and S1-IND-07-A [our strain]). Both *estB* and *astA* genes have been reported in enterotoxigenic E. coli strains in both diseased pigs and pigs with diarrhea, while *pic* and *sat* have been found in enteroinvasive E. coli strains (20–23). Therefore, the presence of novel serotype variants and virulence factors and the association with a wide range of hosts in *E. ruysiae* suggests that this species may have pathogenic and zoonotic potential that requires close monitoring.

### IncB/O/K/Z plasmid characterization.

**FIG 2:**
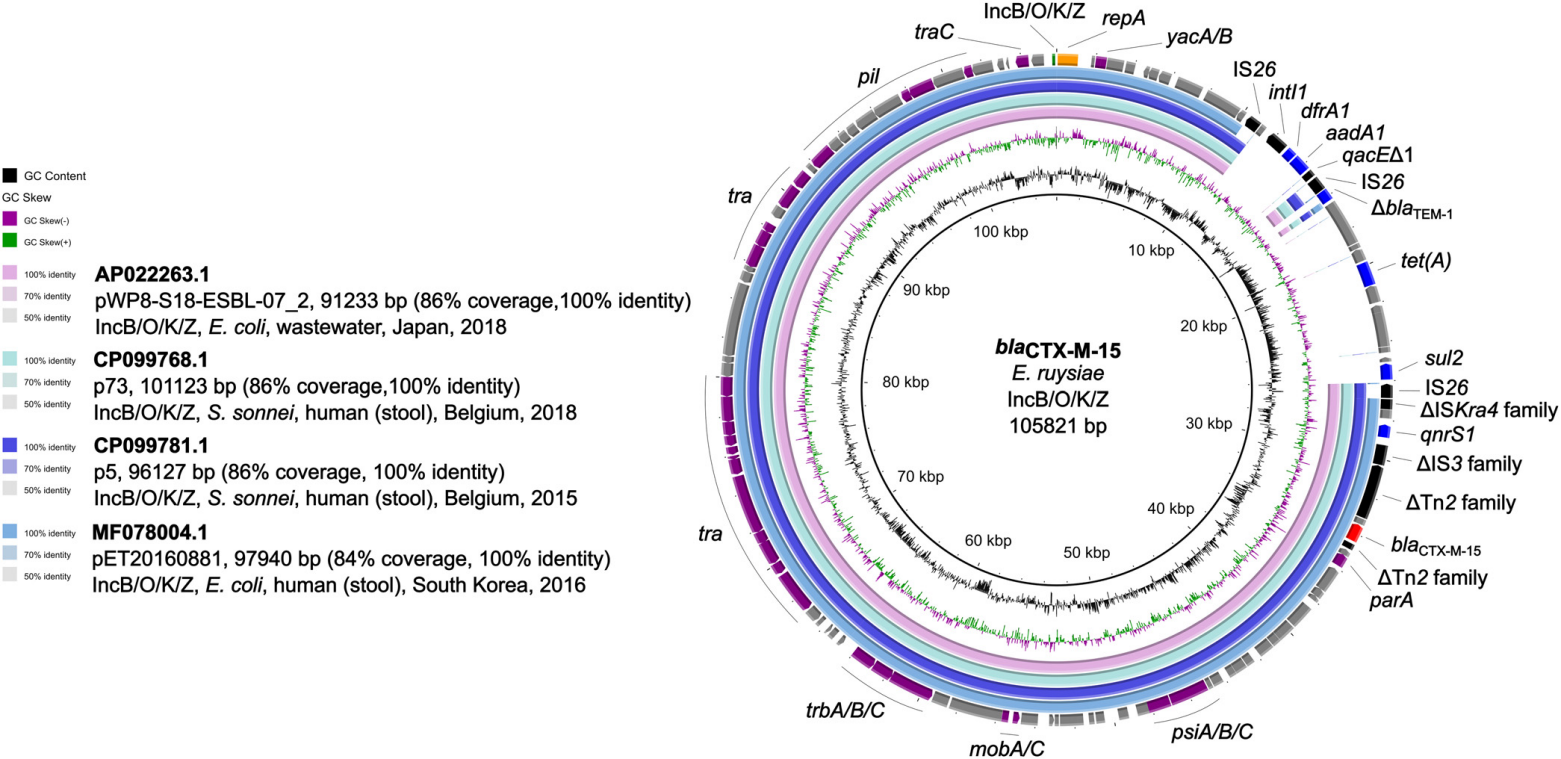
Circular BLASTn comparison of *E. ruysiae* p1-S1-IND-07-A (GenBank accession number CP112984.1) to other similar plasmids of the IncB/O/K/Z replicon type. Sequence similarities to p1-S1-IND-07-A (center) are represented by colored rings. GenBank accession numbers, plasmid name and size, BLASTn coverage and identity, replicon type, plasmid host, isolation source, country, and year of isolation are shown on the left for each comparison. Plasmid genomic features of interest are annotated according by color: red (*bla*_CTX-M-15_), blue (ARGs), black (integron and IS elements), purple (plasmid maintenance and conjugation-related genes), gray (other coding genes). A delta (Δ) symbol corresponds to a partial/incomplete gene. See Table S5 for the gene annotation coordinates shown.

In our *E. ruysiae* strain, *bla*_CTX-M-15_ and five other ARGs were associated with a unique IncB/O/K/Z plasmid named p1-S1-IND-07-A (Fig. 2; Table S1). This plasmid was not present in the other *E. ruysiae* strains analyzed in this study (Fig. 1). However, p1-S1-IND-07-A was similar to plasmids carried by E. coli and Shigella sonnei strains (100% identity and 84% to 86% coverage). Compared to p1-S1-IND-07-A, the other plasmids were missing an IS*26* flanking region containing *tet*(A) and *sul2* and including a disrupted class I integron (missing *sul1*) region associated with the *dfrA1*-*aadA1*-*qacE*Δ1-IS*26*-Δ*bla*_TEM-1_ cassette. Similar integron regions have been reported in hyperepidemic carbapenemase-producing E. coli strains also carrying *bla*_TEM-30_-associated IncI1 plasmids from colonized employees of a Swiss veterinary clinic (24).

Plasmids of the IncB/O/K/Z replicon type are conjugative and associated with a variety of ARGs (e.g., *bla*_CTX-M_) and multiple hosts (e.g., E. coli, S. sonnei, and Klebsiella pneumoniae) (25–27). In fact, p1-S1-IND-07-A displayed a high conjugation efficiency of 4.34 × 10^−2^ with E. coli J53d-R1 (Table 1), thus confirming that not only can *E. ruysiae* acquire p1-S1-IND-07-A but it can also transmit it to other *Enterobacterales* species.

### Conclusions.

International travel and living in high regions of endemicity increase the risk of acquiring emerging MDR-*Ent* in the gut, which may include zoonotic diseases that can be transmitted between humans, animals, and the environment (28). Species with such zoonotic potential are of concern because they can silently colonize human and animal intestinal tracts.

In the present work, we showed that *E. ruysiae*, which is mainly found in animals and the environment, has the ability to acquire worrisome ARGs (e.g., *bla*_ESBL_) and transfer them to other clinically important bacteria. Therefore, the application of WGS surveillance methods (e.g., species ID and SNV analysis) is essential for the discovery and characterization of novel Escherichia species with zoonotic potential and the ability to serve as reservoirs for resistance plasmids. In this context, it is crucial to develop and improve routine laboratory identification techniques such as MALDI-TOF MS to distinguish between Escherichia species.

## MATERIALS AND METHODS

### Stool screening.

A healthy 62-year-old Swiss man living in India for 1.3 years was screened for intestinal carriage of multidrug-resistant *Enterobacterales* (MDR-*Ent*) on his return to Switzerland in 2022 as part of an ongoing prospective screening study (https://data.snf.ch/grants/grant/192514). The stool sample was collected by the volunteer in a sterile container and sent within 1 day to the Institute for Infectious Diseases in Bern for processing (see below). No hospitalization or diarrhea was reported, but previous antibiotic use (not specified) was noted in two independent episodes 2 and 4 months before arrival in Switzerland. The study participant provided written consent, and the study was approved by the ethical committee of the Canton Bern, Switzerland (2020-01683).

A stool sample (~50 μg) was preenriched for 6 h in 10 mL Luria-Bertani (LB) broth supplemented with cefuroxime (30-μg disk; BD BBL Sensi-Disc). The preenrichment was then diluted to a 0.5 McFarland standard, and a 100-μL aliquot was plated overnight at 36 ± 1°C on a CHROMID ESBL agar plate (bioMérieux). The resulting colonies were subcultured on MacConkey II agar (MAC; BBL, Becton, Dickinson) overnight at 36 ± 1°C for further processing.

### Species identification and antimicrobial susceptibility testing.

Species identification of overnight-grown colonies was conducted using MALDI-TOF MS (Bruker; FlexControl v3.4 [build 135.14], MBT Compass v4.1 [build 100], BDAL RUO library 12 [11,897 mass spectral profiles {MSPs}]). Antimicrobial susceptibility testing (AST) was performed by broth microdilution implementing the Sensititre GNX2F panel (Thermo Fisher Scientific). Results were interpreted according to the 2023 European Committee on Antimicrobial Susceptibility Testing (EUCAST) v13.0 breakpoints for *Enterobacterales* (https://www.eucast.org/clinical_breakpoints).

### Whole-genome sequencing.

Genomic DNA for WGS was extracted from strain S1-IND-07-A using the Invitrogen PureLink microbiome DNA purification kit (Thermo Fisher Scientific), and the quality was assessed using NanoDrop and Qubit 3 (Thermo Fisher Scientific). Short-read WGS was performed on a NovaSeq 6000 platform (Illumina; 2 × 150-bp paired-end output), while long-read WGS was conducted on a MinION sequencer (Oxford Nanopore) using a rapid barcoding library prep (SQK-RBK004) and a FLO-MIN 106D R9 flow cell, as described previously (29–31). In brief, short and long reads were preprocessed to remove sequencing adaptors using Trimmomatic v0.36 (https://github.com/usadellab/Trimmomatic) and Porechop v0.2.4 (https://github.com/rrwick/Porechop), respectively. Long reads were filtered by read quality using Filtlong v0.2.1 (https://github.com/rrwick/Filtlong) (parameters: minimum length, 1,000 bp; target bases, 1 billion). A complete genome assembly was generated using the hybrid pipeline from Unicycler v0.4.8 (https://github.com/rrwick/Unicycler), and its completeness (i.e., circular genome) was confirmed with an independent long-read-only assembly using Flye v2.9-b1768 (https://github.com/fenderglass/Flye) (parameters: “–nano-raw” and “–iterations 5”). Genome assembly coverage was calculated using QualiMap v2.2.2-dev (http://qualimap.conesalab.org/).

### Genome assembly and characterization.

The genome assembly of S1-IND-07-A was screened for ARGs, plasmid replicon sequences, sequence type (ST), core genome ST (cgST), serotype, and virulence using ResFinder v4.1, PlasmidFinder v2.1 (parameters: 60% minimum coverage and 50% minimum identity), MLST v2.0 (parameter: E. coli number 1 configuration), cgMLSTFinder v1.2 (parameter: E. coli [Enterobase]), SerotypeFinder v2.0, and VirulenceFinder v2.0 (parameters: 60% minimum coverage and 95% minimum identity), respectively, from the Center for Genomic Epidemiology (https://www.genomicepidemiology.org/). In addition, the phylogroup was determined using the ClermonTyping tool (Clermont Type) available at Enterobase (https://enterobase.warwick.ac.uk/) (Escherichia/*Shigella* database). Finally, the species ID was confirmed using the Type (Strain) Genome Server (TYGS) (https://tygs.dsmz.de/) and by ANIb (based on BLAST) using JSpeciesWS (https://jspecies.ribohost.com/jspeciesws/) with the genome assembly.

The genome assemblies were automatically annotated using the NCBI Prokaryotic Genome Annotation Pipeline. Insertion sequences (ISs) were manually confirmed using ISfinder (https://isfinder.biotoul.fr/) with the BLASTn algorithm. Circular BLASTn plasmid comparisons were performed using BLAST Ring Image Generator v0.95 (https://brig.sourceforge.net/) with the most similar plasmids retrieved from NCBI BLASTn (https://blast.ncbi.nlm.nih.gov/Blast.cgi) and PLSDB v2021_06_23_v2 (https://ccb-microbe.cs.uni-saarland.de/plsdb/) on 27 January 2023.

### Database search and core genome phylogeny analysis.

*E. ruysiae* genomes were retrieved from the NCBI (query, assembly = “Escherichia ruysiae”) and Enterobase (https://enterobase.warwick.ac.uk/species/index/ecoli) (query, data type [Clermont Type {ClermonTyping}] = “cladeIII” and “cladeIV”) databases on 30 January and 2 March 2023, respectively. The species IDs of all the retrieved genomes were confirmed using the TYGS tool as described above.

A core genome alignment was performed using Snippy v4.4.5 with strain OPT1704 (GenBank assembly accession number GCA_902498915.1) as the mapping reference. Genome regions with recombination were detected and removed using Gubbins v2.3.4 (https://github.com/nickjcroucher/gubbins). Furthermore, IS regions were identified with ISEScan v1.7.2.3 (https://github.com/xiezhq/ISEScan) and subsequently used to mask single nucleotide variants (SNVs) localized within these regions (see Table S3 in the supplemental material). A maximum likelihood core genome phylogeny (unrooted) was inferred using IQ-TREE v2.1.2 (http://www.iqtree.org/) with the general time reversible (GTR) model with ascertainment bias correction (parameters: GTR + ASC), 1,000 ultrafast bootstraps (UFBoot; parameter: “-bb”), and the SH-aLRT test (parameter: “-alrt”). The tree was visualized and annotated using iTOL v6.7.2 (https://itol.embl.de/). Unless otherwise noted, all software analyses described above were performed with default parameters.

### Conjugation experiments.

The rifampicin-resistant E. coli recipient strain J53d-R1 was conjugated with the donor S1-IND-07-A as previously described (30). In short, bacterial conjugation was conducted in liquid LB broth for 16 h at 36 ± 1°C. The resulting transconjugants (TCs) were selected on MAC agar plates supplemented with rifampicin (50 μg/mL) and ampicillin (100 μg/mL). Phenotypic and genotypic confirmation of TCs was performed by broth microdilution as described above and by PCR using previously published *bla*_CTX-M_ group I primers (32). Experiments were performed in duplicate.

### Data availability.

The complete genome sequence of S1-IND-07-A has been deposited at GenBank (accession numbers CP112983 to CP112985) and under BioProject accession number PRJNA903117.
